# New Trends in Antioxidant Compounds: A Precise Nutraceutical in Cardiometabolic Disorders

**DOI:** 10.1155/2019/4794563

**Published:** 2019-05-13

**Authors:** Cristiana Caliceti, Norifumi Urao, Paola Rizzo, Mariateresa Giuliano

**Affiliations:** ^1^Department of Chemistry “Giacomo Ciamician”, University of Bologna and Istituto Nazionale Biostrutture e Biosistemi (INBB), Rome, Italy; ^2^Department of Pharmacology, Center for Cardiovascular Research and Center for Wound Healing and Tissue Regeneration, Department of Kinesiology and Nutrition, University of Illinois at Chicago, Chicago, IL, USA; ^3^Department of Morphology, Surgery, and Experimental Medicine, Laboratory for Technologies of Advanced Therapies (LTTA), University of Ferrara and Maria Cecilia Hospital, GVM Care & Research, Cotignola, Italy; ^4^Department of Experimental Medicine, TESLab University of Campania Luigi Vanvitelli, Naples, Italy

Cardiometabolic disorders are among the leading causes of morbidity and mortality in the western world. The most cost-effective preventive approach still remains a personalized diet and physical activity, but novel therapeutic approaches are still needed to delay the progression of these conditions more efficiently compared to existing treatments. As shown with other pathologies, it is becoming increasing clear that a “precise and personalized” strategy holds more possibilities of success compared to traditional approaches. Most medical treatments are designed for the “average patient” as a “one-size-fits-all-approach,” which may be successful for some patients but not for others. Precision medicine is an innovative approach to tailor disease prevention and treatment that takes into account differences in genetic background, environment, and lifestyles. The goal of precision medicine is to administer the right treatments to the right patients at the right time.

Research on food bioactive molecules represents an emerging strategy to evaluate the role of functional foods and supplements in health and disease prevention. There is well-established evidence of the pharmacological properties of micronutrients that render them therapeutically effective in chronic inflammatory diseases. Although caution should be exercised in using antioxidant supplementation, antioxidant foods as dietary components play an important role in the management of cardiometabolic disorders. There is documented evidence of disease-modifying effects of nutritional compounds with anti-inflammatory and antioxidant effects. These compounds have specific applications in ameliorating oxidative stress-induced inflammatory diseases such as diabetes mellitus and cardiovascular diseases. However, due to the limited number of studies, the role of many of these supplements in chronic disease prevention is still unclear. Observational studies have suggested that foods such as fruits and vegetables, nuts, chocolate, and fatty fish, as well as beverages such as tea, wine, and coffee, are associated with a wide range of health benefits. As a result, many have postulated that various bioactive molecules and/or nutrients in these foods may be responsible for the observed health-related effects. Yet annual sales of dietary supplements continue to rise in the US, Europe, and Asia. This may be explained in part by the perception that supplements containing bioactive molecules and nutrients help ensure an adequate intake not only to prevent deficiency of essential vitamins and minerals but also to potentially reduce the risk of major chronic diseases.

In this issue, G. Aquila et al. review the effects of widely used nutraceuticals on the Notch pathway, a major regulator of the functions of endothelial cells and macrophages, the predominant cell types involved in cardiometabolic disorders. The knowledge of the type of modulation, if any, of the Notch pathway by specific compounds could help to identify the most effective nutraceutical, based on its effect on Notch, for a specific individual and/or patient. Similarly, the review article by J. Lietava et al. on the activities of Cornelian cherries at different steps of atherosclerosis provides useful information of the best time for intervention with this compound. Precise and personalized treatments are possible only by dissecting the molecular mechanisms modulated by a specific agent. In this issue, J. Tian et al., by showing that Ginkgo biloba restores autophagy through the mTOR pathway, provide new insights on the well-known protective action of this nutraceutical in the context atherosclerosis, whereas M. Ucci et al. report novel aspects of *β*-carotene and lycopene-mediated protection of vascular endothelial cells under diabetic conditions. Lastly, C. Caliceti et al. identified the specific peptides from cauliflower leaves able to protect vascular cells, thus providing a further example of “precision” approach to obtain increased yield of the specific, bioactive compounds from agriculture waste. Given the growing social and environmental interest for the efficient reuse of agriculture waste co- and by-products, often rich in bioactive compounds, the identification and recovery of such compounds represent an efficient strategy to improve the ecosustainability of cultivations by reducing waste disposal.

